# Thrombosis of the vasa vasorum of the large and medium size pulmonary artery and vein leads to pulmonary thromboembolism in COVID-19

**DOI:** 10.4322/acr.2024.491

**Published:** 2024-05-22

**Authors:** Hubert Daisley, Oneka Acco, Martina Daisley, Dennecia George, Lilly Paul, Errol James, Arlene Rampersad, Farhaana Narinesingh, Ornella Humphrey, Johann Daisley, Melissa Nathan

**Affiliations:** 1 General Hospital, San Fernando, Trinidad and Tobago; 2 The University of the West Indies, Mona, Jamaica; 3 Princess Alexandra Hospital, The Valley, Anguilla; 4 Scarborough General Hospital, Signal Hill, Trinidad and Tobago; 5 Beacon Plastic and Cosmetic Surgery, Woodbrook, Port of Spain, Trinidad and Tobago; 6 The University of the West Indies, St Augustine, Trinidad and Tobago

**Keywords:** COVID-19, Pulmonary Embolism, Vasa Vasorum, Thromboembolism

## Abstract

The vasa vasorum of the large pulmonary vessels is involved in the pathology of COVID-19. This specialized microvasculature plays a major role in the biology and pathology of the pulmonary vessel walls. We have evidence that thrombosis of the vasa vasorum of the large and medium-sized pulmonary vessels during severe COVID-19 causes ischemia and subsequent death of the pulmonary vasculature endothelium. Subsequent release of thrombi from the vasa interna into the pulmonary circulation and pulmonary embolism generated at the ischemic pulmonary vascular endothelium site, are the central pathophysiological mechanisms in COVID-19 responsible for pulmonary thromboembolism. The thrombosis of the vasa vasorum of the large and medium-sized pulmonary vessels is an internal event leading to pulmonary thromboembolism in COVID-19.

## INTRODUCTION

The pandemic of SARS-CoV-2 began in December 2019 in Wuhan, China, causing a disease spectrum referred to as COVID-19.^1^ No country has been spared the astounding morbidity and mortality of this disease. Based on the excess mortality estimates, WHO has determined that there were more than 3.4 million COVID-19 deaths in 2020.^2^ In Trinidad and Tobago from the 3^rd^ of January 2020 to the 18^th^ of January 2023, there were 186,685 confirmed cases of COVID-19, with 4,297 deaths reported to WHO.^3^

The SARS-CoV-2, the etiological agent responsible for COVID-19, is relatively unstable and has mutated several times. There have been numerous viral sub-variants, including the Alpha, Beta, Delta, Gamma, and Omicron variants. Comparing the alpha, beta, and delta variants, the Omicron variant is the least pathogenic.^4^ At present, the JN.1 strain is a sub-variant of omicron in circulation.^5^

There are still debates concerning the pathogenesis of severe acute respiratory syndrome coronavirus 2 (SARS-CoV-2). A growing body of studies suggests a pivotal role for a dysregulated or exacerbated immune response against SARS-CoV-2, leading to an intense inflammatory response. This dysregulated immune inflammatory response causes the release of pro-inflammatory cytokines, which promote the production of reactive oxygen species (ROS) that cause stress and cell damage at the systemic level,^6^ primarily affecting the lungs.^7^

Angiotensin-converting enzyme 2 (ACE-2) receptor sites are the targets for the SARS-CoV-2 virus. These receptor sites are located on nasal and oral mucosae, nasopharyngeal cells, enterocytes, type I and type II epithelial cells in the alveolus, and endothelial cells and pericytes^8^ in the microcirculation. Severe acute respiratory syndrome coronavirus 2, (SARS-CoV-2) is transmitted through respiratory droplets and aerosols, with an incubation period of 4–5 days. Although the infection is symptomless in some cases, most patients present with mild to moderate respiratory disease, experiencing cough, fever, headache, myalgia, and diarrhea. The severe illness usually begins approximately one week after the symptoms’ onset. The most common symptom of severe disease is dyspnea, which is a result of hypoxemia. Soon after the onset of dyspnea and hypoxemia, a progressive respiratory failure develops in patients with severe COVID-19.^9^

The vaccines have provided some amelioration of the disease.

Although all tissues and organs are affected, the lung involvement of the SARS Cov-2 results in severe acute respiratory disease. Thrombosis of the microvasculature within the lungs, which Includes the vasa vasorum of the major pulmonary vessels, sets the stage for major pulmonary pathology.^8,10-12^ In this study, attention was paid to the pulmonary pathology in severe COVID-19 that resulted in death.

## MATERIAL AND METHODS

We conducted autopsies on three patients who had a SARS-CoV-2 infection and died at home and were not submitted to ventilator assistance. The patients were all male with ages 45 years, 68 years and 63 years. Three suffered from diabetes mellitus, and one had stage 4 prostate cancer. The infection was confirmed at autopsy with a reverse transcriptase polymerase chain reaction (RT-PCR) performed on a nasopharyngeal swab sample.

All of the autopsies were performed by one pathologist alone without any assistance using standardized technique. All of the necessary precautionary measures to protect and prevent infection were taken during the external and internal portions of each autopsy. Dissections of the deep leg veins were performed in all cases, and there was no evidence of thrombi.

The average weight of each lung was 700 g, and were consolidated. Tissue samples were fixed in a 10% formalin alcohol mixture and processed routinely.

## RESULTS

Histopathological sections of all lung samples from the three cases in the study were prepared and stained with H&E and periodic acid Schiff.

**Figure 1:**
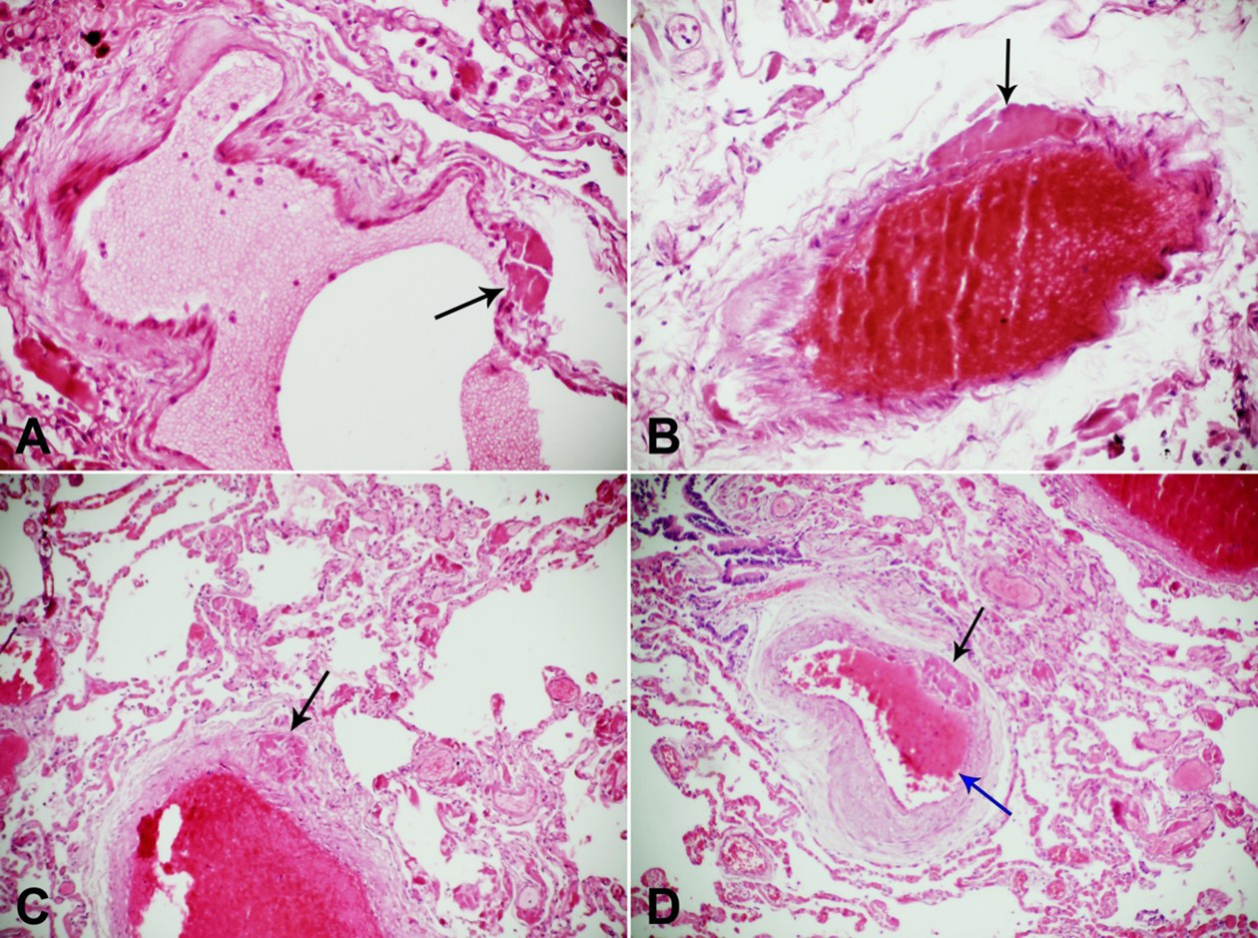
Photomicrograph of the lung. **A –** thrombosis of the vasa interna (black arrow) of a pulmonary vein in COVID-19 (H&E 10x20); **B –** thrombosis of the vasa interna (black arrow) of a pulmonary artery with thrombosis of the pulmonary artery, in COVID-19(H&E 10x20); **C –** thrombosis of the vasa externa (black arrow) and fibrin thrombus within the lumen in COVID-19&E 10x20); **D –** thrombosis of a medium size pulmonary artery with thrombosis of its vasa interna (black arrow) and new thrombus formation within the lumen (blue arrow) in COVID-19 (H&E 10x20).

**Figure 2:**
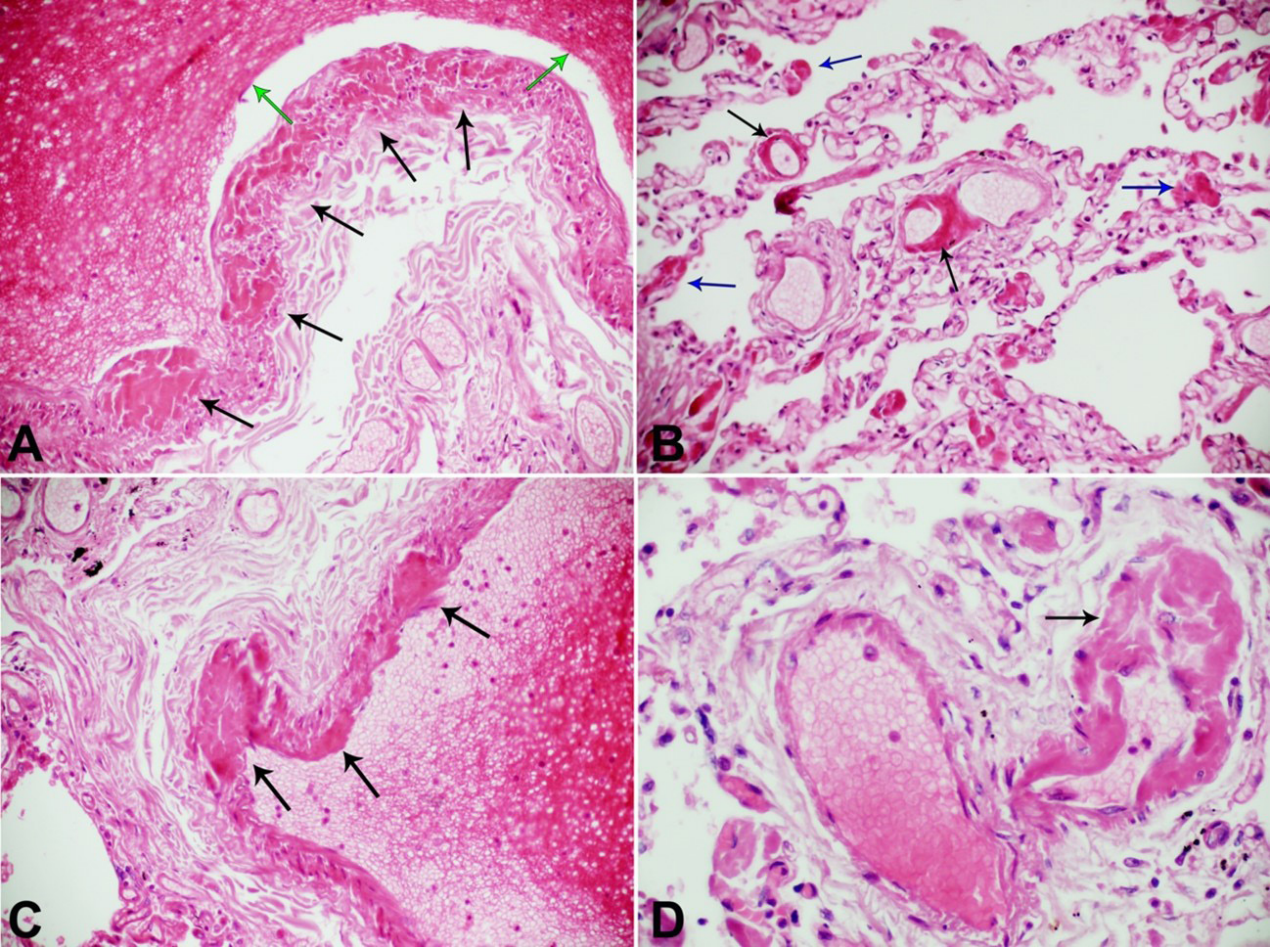
Photomicrograph of the lung. **A –** multiple vasa interna thrombosis (black arrows) in the wall of a pulmonary artery, and fibrin thrombus in its lumen of the pulmonary artery (green arrow) in COVID-19 (H&E 10x20); **B –** thrombosis of arterioles (black arrows) and capillaries (blue arrow) of the lungs in COVID-19 (H&E 10x20); **C –** thrombi in the vasa vasorum interna (black arrows) and fibrin thrombus in the lumen of the pulmonary artery, COVID-19 (H&E 10x20); **D –** thrombosis of venule (black arrow) in COVID-19 (H&E 10x20).

**Figure 3:**
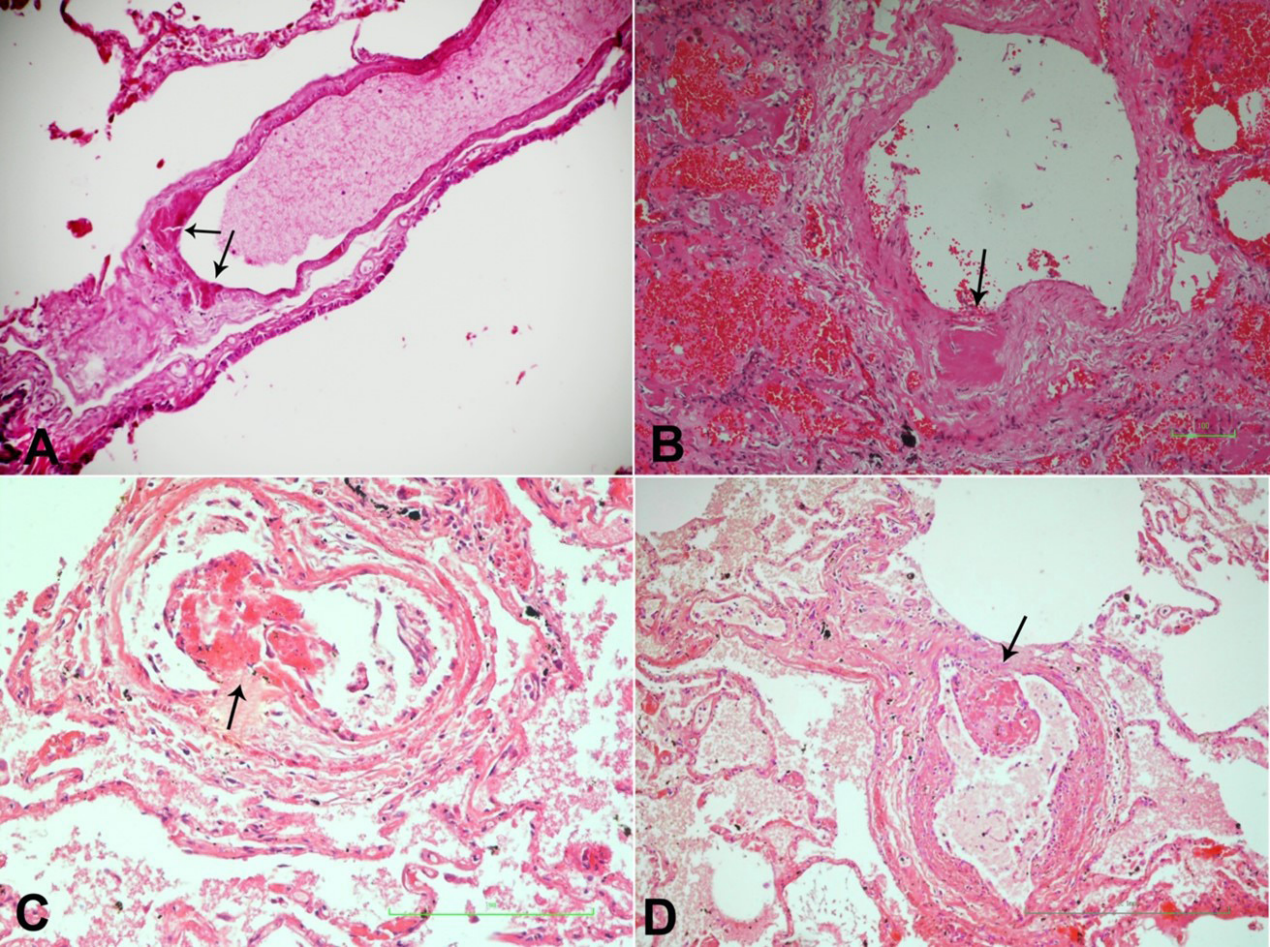
Photomicrograph of the lung. **A –** thrombosis of vasa interna of pulmonary vein in COVID-19 (black arrow)(H&E 10x20); **B –** thrombosis of the vasa interna (black arrow) of a pulmonary artery in COVID-19 and prostate cancer. There is also pulmonary hemorrhage (H&E 10x40); **C –** thrombosis of vasa interna (black arrow) of pulmonary artery in COVID-19 with impending embolism (H&E 10x40); **D –** thrombosis of vasa interna (black arrow) of the pulmonary artery in COVID-19 with impending embolism (H&E 10x40).

Histological examination of the lungs provided the most prominent pathology. Fibrin thrombi deposits were seen in alveolar capillaries, pre-capillary arterioles, pre-capillary venules, and collecting venules. Extension of fibrin deposits into alveolar spaces from injury to the endothelium gave rise to lung consolidation. This fibrin alveolar exudate was not associated with inflammatory cells. Hence, at this acute exudative stage, true pneumonia was not present. Thrombosis of the vasa vasorum interna and externa of the large, medium, and small pulmonary arteries and veins were evident. Thrombosis of the bronchial capillaries and venules was also evident. Thrombosis of pulmonary arteries and veins of varying sizes was also evident. Focal alveolar fibrosis was also evident. (Figures 1, 2 and 3).

## DISCUSSION

We demonstrated the pathological findings of the COVID-19 lung, as seen in the figures above. These findings were not confused with the pathology of the ventilator lung, which formed the basis of earlier studies of COVID-19 pulmonary disease, since many of those cases were hospitalized and had ventilator assistance.^11,13^ All of our cases in this study died at their homes and had no ventilator assistance.

Pulmonary pathology contributed to the major cause of death in each case, with thrombosis of capillaries, venules, arterioles, vasa vasorum of pulmonary vessels (? thrombotic microangiopathy), and pulmonary arteries and vein thromboembolism having fatal consequences.^14-16^

The association of pulmonary embolism with deep vein thrombosis in COVID-19 remains unclear. A growing body of evidence supports pulmonary thromboembolism in COVID-19 as an internal pulmonary event. In one study, more than 50% of the patients with pulmonary embolism lacked deep vein thrombosis.^16^

De Cobelli et al.^17^ supported the hypothesis of a pathogenic relationship between COVID-19 lung inflammation and pulmonary vascular thrombosis and challenged the previous definition of pulmonary embolism associated COVID-19 pneumonia. Mandal et al.^18^ shared their experience that in-situ pulmonary artery thrombosis occurred in COVID-19. It is a growing knowledge that a hypercoagulable state develops in COVID-19 and that immunothrombosis is the main pathophysiological result that significantly contributes to COVID-19-associated pulmonary thrombosis.^19-23^

Pulmonary vein thrombosis^24,25^ has been reported to occur in COVID-19, which further confirms that pulmonary thromboembolism is more of an internal pulmonary event. SARS-CoV-2 causes an endotheliitis in the microcirculation.^26^ Pericytes are the cells in the lung microcirculation that are in intimate contact with the endothelial cells, covering approximately 60–70% of the abluminal endothelial cell surface, and together with the endothelial cells play a major role in the lung pathophysiology of COVID-19.^8,27-29^

The microcirculation within the lungs viz the alveolar capillaries, the pre-capillary arterioles, pre-capillary venules, and collecting venules, the microvasculature of the bronchial circulation^29-31^ and vasa vasorum, of pulmonary arteries and veins are all involved in the pathogenesis of severe COVID-19. Endotheliitis caused by either attachment of SARS-CoV-2 S-protein to ACE2 sites on microvascular endothelial cells and pericytes, or cytokine release during this attachment(s), causes injury of the endothelium,^6^ with exudation of fibrin and the formation of fibrin thrombi within the microcirculation. The pulmonary microvascular pericytes have gained prominence as the major involved cell, mediating vascular inflammation and thrombosis in SARS-CoV-2 pulmonary infection.^8,32-34^ Thrombosis of the vasa vasorum^8,10^ of the medium and large pulmonary arteries and veins, together with thrombosis of these pulmonary vessels, also occur; see figures above.

The vasa vasorum are a network of microvessels within the walls of large and medium arteries and veins with importance to the overall health of these “host” vessels. The arterial vasa vasorum externa deliver oxygen and nutrients to the parent vessel, and venous and lymphatic vasa vasorum interna, which remove waste and extracellular fluid to adjacent veins and lymphatic vessels, respectively.^35^

The vasa vasorum of the pulmonary arteries and veins are involved in the pathogenesis of COVID-19.^8,10,36,37^ Thrombosis of the vasa externa of the large and medium-size pulmonary vessels in COVID-19, see figures above, causes ischemia and subsequent death of pulmonary vascular endothelium, with release of the thrombus from the vasa interna into the pulmonary circulation (Figure 3). Thrombosis of the pulmonary vessels may also occur at the site of the ischemic injured pulmonary endothelial cells, and as a result of the hypercoagulable state that exists in COVID-19.^38,39^ These mechanisms of thromboembolism are the primary event leading to pulmonary artery and vein thromboembolism in COVID-19. This thrombosis initiates with the involvement of the microvasculature of the vasa vasorum interna and externa, shown in the above figures. This mechanism of the internal pulmonary thrombosis may be the predominant pathophysiology in severe COVID-19 pulmonary thromboembolism, although deep vein thrombosis also contributes.^40^

## CONCLUSION

We have demonstrated that in severe COVID-19, thrombosis of the vasa vasorum of the medium-sized and large pulmonary arteries and veins is a major pathological event leading to pulmonary thromboembolism in COVID-19. Pulmonary artery and venous thromboembolism in COVID-19 is an internal pulmonary event that occurs because of thrombosis of the vasa vasorum of the large and medium-sized pulmonary vessels.
